# Exploring the Social and Cultural Influences on Advance Care Planning Engagement for Patients Living With Cancer: A Hermeneutic Phenomenology Study

**DOI:** 10.1111/nin.70109

**Published:** 2026-05-17

**Authors:** Cheng‐Pei Lin, Chih‐Yun Huang, Jen‐Kuei Peng, Hsiao‐Ting Chang

**Affiliations:** ^1^ Institute of Community Health Care, College of Nursing National Yang Ming Chiao Tung University Taipei Taiwan; ^2^ Cicely Saunders Institute of Palliative Care, Policy and Rehabilitation, Florence Nightingale Faculty of Nursing, Midwifery, and Palliative Care King's College London London UK; ^3^ Department of Family Medicine, College of Medicine National Taiwan University Taipei Taiwan; ^4^ Division of Family Medicine, Department of Family Medicine Taipei Veterans General Hospital Taipei Taiwan; ^5^ Faculty of Medicine, School of Medicine National Yang Ming Chiao Tung University Taipei Taiwan

**Keywords:** advance care planning, cancer patients, hermeneutic phenomenology, Leininger's Theory of Culture Care, social and cultural factors

## Abstract

Guided by Leininger's Theory of Culture Care, this hermeneutic phenomenological study explored how social and cultural contexts shape engagement in advance care planning (ACP) among people living with cancer in Taiwan. Semi‐structured interviews were conducted with 26 stakeholders, including 8 patients, 7 family caregivers, and 11 healthcare providers, recruited from oncology and ACP outpatient clinics, inpatient wards, and home care services at a tertiary medical center. Data were audio‐recorded, transcribed verbatim, and analyzed using Naeem's six‐step approach. Six interrelated influences on ACP engagement were identified: socioeconomic circumstances, prior experiences and personal background, religious beliefs and life values, relational dynamics with family and significant others, wider social norms, and political and legal requirements. These factors shaped whether ACP was seen as acceptable, when it was introduced, who was involved in decision‐making, and how discussions were negotiated within families and with healthcare professionals. The findings show that ACP engagement in Taiwan is a relational, culturally embedded process. Culturally congruent, nurse‐led ACP approaches that address relational, socioeconomic, and legal structural influences are needed to support family‐centered cancer care.

## 1 Introduction

Advance care planning (ACP) plays a critical role in enhancing the quality of end‐of‐life care by empowering individuals to articulate their preferences and values regarding future medical treatment (Mori et al. 2025; Sudore et al. 2017). However, the implementation and effectiveness of ACP are profoundly influenced by cultural, religious, and societal contexts (McDermott and Selman 2018). As such, a one‐size‐fits‐all model, predominantly based on Western paradigms, may fail to resonate with multicultural and non‐Western populations (Lin et al. 2019). Recognizing the pivotal role of multiculturalism in ACP is not only an ethical imperative for inclusivity but also a practical necessity for nursing in delivering meaningful, person‐centered cancer care. This perspective is supported by a consensus among international experts in Asia, who emphasize the importance of culturally sensitive, family‐centered approaches in ACP (Mori et al. 2025).

Cultural beliefs and values may shape how individuals and families perceive death, decision‐making autonomy, and engagement in ACP. In some Asian contexts, values such as filial piety, family harmony (Mori et al. 2023), and relational autonomy (Foo et al. 2024) may influence the timing, approach, and perceived acceptability of ACP conversations. Within such contexts, some patients may prefer shared or family‐centered decision‐making rather than an exclusively individual model (Mori et al. 2023). Likewise, in some religious or Indigenous communities, spiritual beliefs and broader worldviews may influence whether and how people engage in discussions about future serious illness or end‐of‐life care (Lillie et al. 2020; Martina et al. 2022a, 2022b; Sinclair et al. 2014). Rather than implying fixed cultural patterns, these findings highlight the importance of approaching ACP in ways that are sensitive to diverse cultural and social contexts.

Consistent with the need to adapt ACP to diverse cultural and social contexts, multicultural sensitivity also extends to communication preferences and linguistic nuance. In addition to shaping values and decision‐making processes, culture and social norms influence how ACP‐related concepts are expressed, interpreted, and emotionally received. Studies have shown that even core ACP terms, such as “readiness” and “end‐of‐life,” require careful cultural and linguistic adaptation to ensure conceptual equivalence and emotional resonance (Liu et al. 2020; Wei et al. 2022). For example, in Japan, “readiness” was translated as *kokoronojyunbi* (mental or spiritual preparation), while “end‐of‐life” was rendered as *jinseinosaisyudankai* (final stage of life), reflecting the importance of culturally congruent phrasing in ACP discussions (Okada et al. 2021). A nuanced understanding of ACP approaches grounded in different social norms is essential for nurses to deliver care with cultural safety and cultural sensitivity (Jonathan et al. 2025).

Building on the importance of cultural beliefs, communication preferences, and linguistic adaptation in ACP, integrating multicultural principles into practice requires action at multiple levels. Relevant strategies include engaging cultural advocates or community‐based health workers, co‐designing interventions with key stakeholders, and tailoring educational materials and resources to reflect the values and needs of the populations served (Lillie et al. 2020; Sinclair et al. 2014). Moreover, successful ACP interventions in multicultural contexts must also take account of wider structural and infrastructural factors, including access to technology, health literacy, and institutional policies, as these conditions can either facilitate or constrain meaningful participation (van Lummel et al. 2023). For example, integrating ACP into routine nursing care supported by telehealth and community‐based services may enhance accessibility and improve responsiveness to the needs of diverse populations (Bange et al. 2024).

Preparing nursing professionals for culturally responsive ACP conversations is essential. This requires competence in culturally appropriate communication, understanding diverse values and preferences, and recognizing the socioecological factors that influence engagement with healthcare and ACP (Jonathan et al. 2025; Martina et al. 2021). Such preparation is important because evidence from non‐Western settings shows that cultural, social, and contextual factors shape the acceptability of ACP, the feasibility of adapted models, the likelihood of ACP discussions, goal‐concordant care, and satisfaction with services (Chikada et al. 2021; McDermott and Selman 2018; Rice and Rhodes 2024; Takenouchi et al. 2024).

Collectively, these findings highlight that attention to multiculturalism is not a peripheral concern, but a foundational component of ethical, effective, and equitable ACP practice. We need to understand the local cultural and contextual nuances before adapting ACP into routine nursing care. Therefore, this study aims to explore the social and cultural influences on ACP engagement among patients living with cancer in Taiwan.

## 2 Methods

### 2.1 Methodological Approach

We conducted a semi‐structured, one‐on‐one qualitative interview research spanning a 14‐month period from March 22, 2022, to May 10, 2023. This study was informed by the principles of hermeneutic phenomenology and theoretically underpinned by Leininger's Theory of Culture Care (Leininger 1991; McFarland and Wehbe‐Alamah 2019), which shaped the research design and the authors' critical interpretive lens in data analysis and reporting.

The reason for adopting hermeneutic phenomenology is to seek a deep, interpretive understanding of the meaning of human beings' lived experience by integrating the study participants' viewpoints, the researchers' preconceptions, and the context in which the experience occurs (Cohen et al. 2000). In this study, we used this approach to explore how patients with cancer experienced participation in ACP with family members and healthcare providers. Data interpretation was situated within the Taiwanese context, including the healthcare system, relevant legislation, service procedures, and prevailing awareness of palliative care and ACP. This focus was informed by evidence suggesting that cultural diversity may influence the uptake and acceptability of ACP (Chikada et al. 2021; McDermott and Selman 2018).

The Cultural Care Diversity and Universality Theory proposed by Madeleine Leininger emphasizes the importance of understanding cultural diversity in nursing care. It is based on the idea that cultural beliefs, values, and practices influence health, wellness, illness, and care practices (Leininger 1991). Leininger categorized the factors influencing care through the Sunrise Model, which includes technological, religious and philosophical, kinship and social, cultural values and lifeways, political and legal, economic, and educational dimensions. These interrelated domains help nurses assess how culture and social structure shape health meanings and guide culturally congruent care (McFarland and Wehbe‐Alamah 2019). Such philosophical understanding of nursing care is aligned with the core value of ACP, which provides patient‐centered and goal‐concordant care (Hadler and Aslakson 2024).

The purpose of adopting Leininger's theory was not to map all relevant factors directly onto the data, but to guide the analysis and illuminate the cultural dimensions shaping ACP experiences and care among patients with cancer. Because healthcare responses are influenced not only by culture but also by evolving social norms and contextual conditions, we used an inductive qualitative analysis informed by Leininger's theory to explore how intersecting cultural and social factors shape ACP experiences in Taiwan.

### 2.2 Settings and Sample

The setting was the ACP outpatient clinics, oncology outpatient clinics, inpatient wards, and home care at a tertiary medical center with around 3000 inpatient beds serving northern Taiwan under the national health insurance scheme. The study medical center was built in 1958 and used to serve only the veterans. Nowadays, it is open to the general public and serves as one of the primary medical centers in northern Taiwan. We adopted purposive sampling for participant recruitment (i.e., patients, their family members and healthcare providers) following the eligible criteria below: (1) patients who were diagnosed with cancer, had participated in an ACP consultation with decisional capacity and could communicate in Mandarin or Taiwanese; (2) family members were appointed by the patient as primary care provider and should accompany the patient during the ACP consultation, and (3) healthcare providers were those at least have 6 month healthcare professional experience, completed the authorized ACP consultation training, and had experience assisting patient in ACP consultation. We excluded people who cannot explicitly express themselves due to mental illness or physical distress, as judged by the clinicians.

Informed by the literature (Zhu et al. 2023), we estimated a sample size of 8–16 participants per group to have adequate information power to answer our research question (Malterud et al. 2016). Given the hermeneutic‐phenomenological design, we intended to invite patients, their family members, and the healthcare professionals who provided the consultation for them, so they could reflect on the same experience and gain a broader, deeper understanding. Given the very challenging nature of involving patient–caregiver dyads in palliative care research (LeBlanc et al. 2013), no patient–family caregiver dyads were required for recruitment.

### 2.3 Data Collection

Guided by the first author, the primary care team (ACP team, oncology team, and home care team) served as gatekeepers and dedicated resources to screen cancer patients for appropriateness to participate in this study based on their disease condition (Preston et al. 2016). Potential eligible patients were referred to the research team for assessing eligibility and explaining the study purpose, as well as answering any questions from patients. The participating patients were invited to appoint one family caregiver who was familiar with their life goals and who was present during the ACP consultation for an interview. The healthcare team providing ACP consultations was invited by the first author directly for an interview. All participants were given at least 24 hr to consider study participation. Those who wished to be interviewed were required to complete a written informed consent form.

Semi‐structured interview topic guides were informed by the literature and discussions within the research team, finalized by piloting with clinical team members (Supplemental Information [Link], [Link], [Link]). Face‐to‐face interviews were conducted, with the option of a virtual conference per participants' preference, due to the pandemic's social distancing policy. For those who chose virtual interviews, we provided a unique meeting link at a time convenient for them and audio‐recorded the entire interview for subsequent analysis. All interviews were conducted by the same researcher, who had training in qualitative research and interviewing. Interview skill coaching was provided by the first author to the researcher to prepare her competence before commencing interviews. The interviews were often conducted right after the patient's ACP consultation. In some cases, it was necessary to conduct interviews at patients' homes or via a virtual conference to accommodate their time schedule or illness conditions.

Field notes were recorded during each interview, followed by completing a reflexivity form by the researcher to reflect on the interview encounter, including study procedure, environment, participants' responses, and interview flow. Monthly meetings with the clinical and research teams were held to discuss any challenges encountered.

### 2.4 Data Processing and Analysis

All interviews were audio‐recorded and transcribed verbatim by the researcher with support from a professional translation agency and scrutinized by the first author. All audio recordings and scripts were stored in an encrypted cloud storage service accessible only to the research team. Data analysis using Naeem et al. (2023) six‐step approach, including (1) familiarizing data and selecting quotations; (2) selecting keywords; (3) coding; (4) developing themes; (5) conceptualizing through interpretation of keywords, codes, and themes, and (6) developing of conceptual model (Naeem et al. 2023). Data analysis began shortly after the first interview was completed, involving the researcher and the first author as the supervisor. The Sunrise Model from Leininger's Theory of Culture Care was used to inform a critical analytical process (Leininger 1991). We expected to identify cultural and social factors unique to the Taiwanese context, so an inductive approach was embedded.

The research team thoroughly reviewed all transcripts, performed initial analysis and coding, and reported preliminary findings at the monthly meeting for feedback from the clinical and research team on possible findings. The findings were later reviewed and refined by the first author as needed. The analysis generated an interpretive account of patients' experiences by integrating the perspectives of all stakeholders within the broader social and cultural context. Member checking was not performed, as evidence reported that it was not beneficial for bias control and would not necessarily improve validity (Birt et al. 2016; Varpio et al. 2017). NVivo 12 was used to organize transcripts and codes during the analysis. We incorporated strategies such as data source triangulation, reflexive diary writing, and iterative discussion among clinical and research teams to enhance study quality and rigor (Carter et al. 2014).

### 2.5 Ethical Considerations

The study received ethical approval from the Hospital Institutional Review Board (IRB No. 2021‐12‐011CC). All participants provided informed consent before participating in interviews.

## 3 Results

Twenty‐six participants, including eight cancer patients, seven family caregivers, and 11 healthcare providers, were interviewed. The interviews averaged 45 minutes (range: 20–60 minutes). The cancer patients had a mean age of 55.5 years. The majority were female, diagnosed with breast cancer, and with a college degree of educational attainment; the family caregivers had a mean age of 46.4 years old. The majority were the patients' children, with a college degree; the healthcare professionals included six physicians, three nurses, one social worker, and one psychologist, with a mean age of 41.8 years. The majority were female with a bachelor's degree (Supplemental Information S4).

Six influences were identified and related to Leininger's Theory of Culture Care by presenting them as context‐specific expressions of the social structure and cultural dimensions:

1. Socioeconomic status, reflecting Leininger's economic factors
2. Prior experiences and personal background, reflecting Leininger's cultural values, lifeways, and educational influences
3. Religious beliefs and life values, reflecting Leininger's religious and philosophical factors
4. Relational dynamics with significant others, reflecting Leininger's kinship and social factors
5. Social norms and expectations, reflecting Leininger's cultural values and lifeways
6. Political and legal requirements, reflecting Leininger's political and legal factors

1. **Socioeconomic status**
  Although Taiwan's National Health Insurance covers healthcare costs for patients, the fees for ACP consultations and AD completion are not included, and an out‐of‐pocket fee (USD 100 = NTD 3500) is charged to the individual. A female patient described this as a factor influencing an individual's willingness to engage in ACP.
  In fact, Taiwan's National Health Insurance (NHI) is considered quite good compared with many other countries. However, at the end of life, some aspects are actually not covered by NHI, including ACP consultation. This will influence people's willingness [in participation].
  (55‐Year‐old female patient)
  Therefore, financial concerns are reported to be a significant influence. Some participants were in favor of completing AD even though an out‐of‐pocket fee was required, as they felt it would provide assurance for their future care. However, for those with limited resources, taking part in ACP and completing AD was a concern due to the financial burden caused by the life‐sustaining treatment and care required to support patients' hospitalization.
  He [patient] said that he felt spending a few thousand New Taiwan Dollars on this, as a way to secure his future care, was very worthwhile. This older gentleman [patient] also mentioned a friend whose family is in a relatively poor financial situation, sometimes struggling to afford each meal. She said, “*Asking her to spend a few thousand dollars on this [ACP consultation] would be unrealistic, as that amount could cover her food expenses for one to two weeks*.”
  (29‐Year‐old social worker)
  To bear such a heavy financial burden just to maintain that kind of life [live with life‐sustaining treatment], I feel it is unnecessary. So, I think this has already become an economic consideration.
  (55‐Year‐old female patient)
  These findings suggest that socioeconomic status shapes access to and engagement in ACP, with out‐of‐pocket costs posing a greater barrier for individuals and families with limited financial resources.
2. **Prior experience and personal background**
  Individuals may be influenced by their prior experiences as patients or caregivers. Some family caregivers reported that previous involvement in caring for a person with cancer increased their openness to engage in end‐of‐life care discussions and planning in advance.
  Because our family has previously cared for a relative with cancer, topics such as death and end‐of‐life care may be less sensitive for us.
  (27‐Year‐old female caregiver)
  An individual's background may also shape engagement in ACP. For example, in Taiwan, some participants were veterans whose values had been shaped by wartime experiences and military service. Their prior exposure to the possibility of death appeared to contribute to greater acceptance of discussions about future end‐of‐life care, as they described themselves as more prepared to confront issues related to death and dying.
  My father [patient] came from mainland China during the war, and I also served in the military, so in some ways our backgrounds are similar. Because of these shared experiences, we may feel less reluctant to discuss end‐of‐life care, perhaps because we see life and death as a natural part of life.
  (59‐Year‐old male caregiver)
  Together, these accounts suggest that prior caregiving experiences and personal background can shape how individuals perceive and engage in ACP.
3. **Religious beliefs and life values**
  In Taiwan, Christianity and Buddhism are among the major religious traditions. Healthcare professionals observed that many participants in ACP consultations held religious beliefs, which may provide comfort and support greater openness to end‐of‐life discussions. At the same time, differing religious understandings of life and death appeared to shape individuals' attitudes toward dying in distinct ways.
  I have encountered many Christians who believed that, during the dying process or at the terminal stage, as long as they were free from suffering, they would eventually return to their heavenly home.
  (49‐Year‐old nurse)
  In my Buddhist faith, life is not defined by its length, but by its meaning. Buddhism has shaped my perspective in this way, and I believe that making decisions in advance is one way of giving life fullness and meaning.
  (63‐Year‐old female patient)
  Religion plays an important and widely accepted role in discussions of life and death in Taiwan, which indirectly influences the patients' ACP engagement. As a multicultural society with diverse religious traditions, Taiwan offers many individuals opportunities to encounter and reflect on death‐related issues through their faith.
  A person's values and beliefs about life, as well as how they view decisions about medical treatments, can all be influenced by faith. Religion may also shape how they prepare for a good death and for the rituals that follow.
  (53‐Year‐old female nurse)
4. **Relational dynamics with significant others**
  Filial piety and social pressure are key barriers. For fear of being judged as unfilial for not “doing everything possible,” and given medical advances that enable life‐sustaining treatment, families often insist on aggressive resuscitation. Consequently, no one dares to take responsibility for stopping or withholding treatment.
  They [the family] felt a strong obligation to pursue all possible measures until the very end and therefore expressed a preference for aggressive resuscitation to the medical team. As one family member recalled, some relatives told her, “*If you do not try to save your father, you are being unfilial. Your father raised you for nothing*.”
  (29‐Year‐old social worker)
  The closeness of family relationships also appeared to influence willingness to engage in ACP. Individuals with weaker family ties were more likely to prefer making decisions for themselves rather than leaving them to family members.
  He [the patient] wished to complete an advance directive because he was not close to these relatives. He did not want decisions concerning his life and death to be made by family members with whom he was unfamiliar, whom he did not particularly trust, or whom he perceived as not genuinely caring about him.
  (34‐Year‐old physician)
  Peer support was also found to shape patients' engagement in ACP in Taiwan. Patients appeared more willing to participate in ACP consultations when accompanied by significant others, such as family members or close friends.
  Peer influence is certainly present. Sometimes, when older patients come for consultation, they say they do not want to suffer the way a certain person did, or that they would prefer to be comfortable like someone else. They also hope to have someone accompany them, so I think peer influence does play a role to some extent.
  (37‐Year‐old female nurse)
  These findings underscore the importance of relational dynamics in shaping ACP engagement. Within families and friendships, expectations of filial responsibility, emotional obligation, and social judgment may constrain open discussion and favor continued life‐sustaining treatment, whereas weaker or more distant relationships may prompt individuals to assert greater autonomy in end‐of‐life decision‐making.
5. **Social norms and expectations**
  In Taiwan, the designation of medical surrogates in ACP discussions for patients with cancer is shaped by both surrogate hierarchies and traditional family roles within prevailing social norms, with the eldest son often expected to assume the surrogate decision‐making role. The quote below illustrates how these traditional role expectations influenced family decision‐making, positioning the eldest son as the primary authority while other family members aligned their views with his.
  The eldest son was expected to assume the traditional family role, shaped by prevailing social expectations and role identities. During the decision‐making process, the younger daughter repeatedly asked her older brother, “*What do you think? How do you feel about this?”* Although she expressed her own views, she continued to seek and defer to her brother's opinion.
  (29‐Year‐old social worker)
  Within an asymmetrical doctor–patient relationship, some participants worried that signing an advance directive (AD) might be seen as undermining the physician's efforts, potentially affecting the therapeutic relationship and subsequent care. Thus, reluctance to complete or disclose an AD reflected not only personal hesitation but also social expectations of respect for medical authority.
  I think that when the attending physician has been very aggressive in pursuing treatment, patients often hesitate to sign this document [AD] and may not want the physician to know they have done so. They may feel apologetic because they see the physician working hard to treat them, and they worry it could be perceived as giving up, which might affect the attentiveness of subsequent care.
  (53‐ Year‐old nurse)
  While death has traditionally been regarded as a sensitive and often avoided topic, some participants described a growing emphasis on patient autonomy and personal choice as increasingly accepted aspects of good clinical practice.
  With social changes over time, people have increasingly come to value their own autonomy and preferred way of living… Palliative care and ACP have also undergone ongoing revision and improvement.
  (60‐Year‐old female patient)
  Engagement in ACP was shaped by social norms surrounding medical authority, death, and decision‐making, and the role of medical surrogates. Some participants described that these norms are gradually changing over time.
6. **Political and legal requirements**

The enactment of the Patient Right to Autonomy Act in Taiwan since 2019 can be understood as a political factor that shapes the process, eligibility, and implementation of ACP and the completion of ADs. Patients reported that the ACP consultation and AD completion process was very rigid and overly complicated. ACP consultations required the presence of family members or close friends as witnesses and could only be arranged at the appointed outpatient clinics when the patient, witnesses, and the healthcare team (i.e., physicians, nurses, social workers, or psychologists) were all available for multidisciplinary discussion.

It seems that you have to make a separate appointment at a specific clinic in order to complete the signing. I wonder whether it might be possible to do it directly during a regular outpatient visit instead. I think there should be more flexibility in how people can choose to complete it, rather than requiring it to be done in only one fixed way.

(57‐Year‐old male patient)

You need someone to accompany you, and you also need two witnesses, so you have to coordinate everyone's schedules. If it has to be a special trip, finding a suitable time is really difficult. Sometimes it also depends on whether family members are available.

(66‐Year‐old female patient)

Although this approach to ACP consultation and AD completion is intended to strengthen the rigor of end‐of‐life decision‐making, it may inadvertently hinder service uptake among patients with cancer.

## 4 Discussion

This study shows that ACP engagement among patients with cancer in Taiwan is not simply an individual decision‐making process, but is shaped by interrelated social, cultural, and structural influences. Interpreted through Leininger's Theory of Culture Care, particularly the Sunrise Model (Leininger 1991), the identified domains reflect several key dimensions of culturally congruent care: socioeconomic status relates to economic factors; prior experiences and personal background to cultural values, lifeways, and educational influences; religious beliefs and life values to religious and philosophical factors; relational dynamics with significant others to kinship and social factors; social norms to broader cultural values and lifeways; and political and legal requirements to structural influences on care. Together, these factors shaped whether ACP was seen as acceptable, when it was discussed, who was involved, and how decisions were negotiated in practice, underscoring that ACP is embedded within the Taiwanese local cultural and healthcare context.

Echoing previous evidence, our findings suggest that ACP interventions can be successfully implemented across diverse populations when tailored to local contexts, cultural beliefs, and care practices. For example, ACP for First Nations populations in Australia, the United States, and Taiwan has emphasized the inclusion of Aboriginal rituals, healthcare workers, and wider community engagement (“Country”) (Chauhan et al. 2025; Goins et al. 2024; Li et al. 2021). In Japan, cultural adaptation of ACP materials has improved uptake (Okada et al. 2021). In Indonesia, attention to religious understandings of suffering and healing has been identified as central to ACP integration (Martina et al. 2022a, 2022b). In Mainland China, optimistic health views and supportive family dynamics could facilitate ACP delivery for cancer patients, while misunderstanding, cultural taboos, and economic burdens were considered barriers (Yan et al. 2025). Such cultural adaptation aligns with Leininger's culture care actions: preserving valued cultural practices, negotiating with local people and systems, and restructuring care to improve acceptance and effectiveness (Leininger 1991).

In our study, political and legal considerations were important influences on ACP engagement (Tu et al. 2026), shaping and interacting with other factors such as the financial requirements for participation and family involvement in decision‐making. In Taiwan, ACP is underpinned by the Patient Right to Autonomy Act since 2019, which provides a formal legal basis for ACP delivery, specifies the consultation process, and gives ADs a legally binding status (Wang 2023). While this framework establishes a clear procedure for healthcare professionals, it also places limits on the participating population, professional roles, and service delivery. For example, family accompaniment during ACP consultation is compulsory, only accredited healthcare institutions with suitably trained staff may provide ACP consultations, and nurses are not permitted to lead ACP consultations independently. An out‐of‐pocket fee for ACP participation is required under this legislation, which is very different from that in other countries. For example, Singapore also has supporting legislation under the Mental Capacity Act 2008 and shares certain cultural similarities with Taiwan, its ACP model differs in terms of eligibility and service delivery without extra fee (Ng et al. 2023). In particular, nurse‐led ACP interventions in Singapore are permitted and have been well received by cancer patients (Yang et al. 2021). However, from May 2025, the Taiwanese government began reimbursing ACP consultation fees for certain patient groups, including older adults with serious illness or multimorbidity, patients with mild dementia, and those with palliative care needs, aiming to improve service uptake and AD completion (National Health Insurance Administration 2025). This illustrates how political change can shape ACP delivery and care.

There is a theoretical concern regarding the application of Leininger's Theory of Culture Care in this study. While Leininger's Theory of Culture Care was foundational in highlighting the importance of culturally congruent care (Leininger 1991), palliative care and ACP now require a broader understanding of culture for better healthcare outcomes (McDermott and Selman 2018; Rice and Rhodes 2024; Takenouchi et al. 2024). In these contexts, culture is not a fixed set of beliefs, but is shaped by local family relationships, the patient's illness experiences, clinician‐patient communication patterns, and social/legal structural influences on decision‐making. Contemporary nursing practice therefore emphasizes cultural humility, critical self‐reflection, and cultural safety, particularly when discussing serious illness issues, future care, and end‐of‐life values. This perspective extends Leininger's theory by promoting shared, family‐centered, and context‐sensitive ACP decision‐making (Mori et al. 2025).

### 4.1 Implications for Nursing Practice and Research

For nursing practice, the findings suggest that ACP for patients with cancer in Taiwan should be approached as a culturally congruent and relational process rather than a one‐time individual decision. Informed by Leininger's theory, nurses should assess how economic conditions, religious and philosophical beliefs, kinship and social relationships, cultural values, and political–legal requirements shape ACP engagement. This includes recognizing financial barriers, family role expectations, prior experiences, and the influence of social norms and medical authority when introducing ACP discussions. Earlier, family‐engaged, and culturally sensitive ACP conversations may better support meaningful decision‐making.

For nursing research, Leininger's theory highlights the need to develop and evaluate ACP models that are responsive to local cultural and social structures rather than relying on decontextualized approaches. Future studies should test nurse‐supported, culturally adapted ACP interventions that address the economic, relational, spiritual, and legal influences identified in this study. Outcomes should extend beyond AD completion to include cultural congruence, family communication, readiness, and decision‐making experience.

### 4.2 Strengths and Limitations

This study has several strengths. First, this is a theory‐driven, qualitative study that utilizes a standardized reporting guideline. Second, we collected diverse viewpoints from stakeholders (i.e., patients, family caregivers, and healthcare providers) to inform a comprehensive understanding of the topic of interest. Third, working closely with the clinical team can increase participants' willingness to take part in this study by fostering rapport among clinicians, patients, and families. Additionally, clinical team members served as gatekeepers and dedicated resources by screening eligible participants and identifying patients whose conditions were relatively stable and who were open to discussing ACP‐related issues, thereby improving recruitment effectiveness (Preston et al. 2016).

Nevertheless, we still faced challenges in recruiting cancer patients and their family members (LeBlanc et al. 2013). This was expected as the general public and healthcare system are not familiar with the concept of ACP due to the newly rolled out legislation. The underpinning legislation of ACP (the Patient Right to Autonomy Act) has been in effect only since 2019, and the concept and development of the service are still in their infancy in Taiwan (Wang 2023). During the study period, 376 individuals completed ACP consultations at the study medical center. Of these, only 22 were cancer patients, and only four agreed to be interviewed. Therefore, we extended our recruitment to include outpatient clinics, inpatient wards, and home care services. Furthermore, the busy schedules of patients with cancer and their family members may reduce their willingness to participate in interviews, as many are in midlife and must balance caregiving responsibilities for both children and older parents alongside work commitments. Lastly, the social distancing policy during data collection influenced participants' willingness to participate in the study, even though we offered a virtual interview option. Therefore, we approached patients and their family members while they were waiting for outpatient appointments, or we visited them at a time and place that was convenient for them, to increase their willingness to participate in the study.

The application of these findings requires careful consideration, as the interviewees were open to discussing palliative care and ACP‐related issues, which may not accurately reflect the broader population. We do not purport to provide an exhaustive rendering of experiences. Rather, these findings contribute to uncovering a sensitive topic previously considered taboo, and that remains insufficiently explored following the rollout of the new legislation and the changing social norms in Taiwan.

## 5 Conclusions

ACP engagement among patients with cancer in Taiwan is shaped by interrelated economic, cultural, relational, and political–legal influences. Viewed through Leininger's Theory of Culture Care, these findings highlight that ACP is not simply an individual act of autonomy, but a culturally and socially situated process. Culturally congruent ACP in this context should therefore be introduced earlier, revisited over time, and conducted in ways that meaningfully engage families and reflect local values, social expectations, and healthcare structures. This may help promote more acceptable, context‐sensitive, and value‐concordant end‐of‐life care.

## Author Contributions

**Cheng‐Pei Lin:** conceptualization, methodology, data analysis and interpretation, writing – original draft, review and editing. **Chih‐Yun Huang:** data collection and curation, data analysis and interpretation, writing – original draft. **Jen‐Kuei Peng:** methodology, data analysis and interpretation, writing – review and editing. **Hsiao‐Ting Chang:** conceptualization, methodology, writing – review and editing.

## Funding

The authors have nothing to report.

## Ethics Statement

The description regarding ethical consideration was reported in the main text.

## Conflicts of Interest

The authors declare no conflicts of interest.

## Declaration of Generative AI and AI‐Assisted Technologies in the Writing Process

Grammarly and ChatGPT‐5 were used for language editing and grammar checking.

## Supporting information

Supporting File 1

Supporting File 2

Supporting File 3

Supporting File 4

## Data Availability

The data that support the findings of this study are available on request from the corresponding author. The data are not publicly available due to privacy or ethical restrictions. All the relevant data are available. The method of data collection and analysis were reported in the main text.
