# Supplementary material for: Exploring the Social and Cultural Influences on Advance Care Planning Engagement for Patients Living With Cancer: A Hermeneutic Phenomenology Study
Source: Nurs Inq. 2026 May 17;33:e70109. doi: 10.1111/nin.70109 (PMC13180463; doi:10.1111/nin.70109)
Supplement: Supplementary file 1 — Supporting File 1 [file NIN-33-e70109-s001.docx]

**Supplemental Information 1. Interview guide for cancer patients**

**Interview Objectives**

1. Feelings and experiences of participating in ACP autonomous decision-making.
2. Factors and considerations influencing the execution of autonomous decision-making (encourage the interviewee to share real experiences and feelings).

**Objective One: Feelings and Experiences of Participating in ACP Autonomous Decision-Making**

- Can you start by sharing the process of your illness?
- How did you come to know about the Patient Autonomy Act/ACP?
- Are end-of-life issues openly discussed at home, or are there any reservations?
- What are your thoughts on ACP and your values about life?
- How did participating in ACP make you feel?
  - Afraid? Because of religious beliefs? Or being unable to let go of someone?
  - Happy? Because you can make decisions for yourself?
  - If you were to share your experiences and feelings about participating in ACP with others, what would you share?
  - At what point did you discuss with doctors and family members?
  - How did participating in ACP change or impact your life?
- Have you heard about the Patient Autonomy Act?
- Based on your experience, how has the Patient Autonomy Act affected your right to medical decision-making? What is your opinion?
  - Do you think the law helps you exercise your medical autonomy?
  - What are the helpful aspects and limitations?

**Objective Two: Factors and Considerations Influencing the Execution of Autonomous Decision-Making**

- What made you want to sign ACP initially?
- Did you discuss with family or friends before signing ACP?
  - What are your family’s thoughts?
  - Who supported you in making the decision?
- Did you encounter any opposition during the discussion and decision-making process?
  - What were the reasons for the opposition?
  - How did you communicate with them?
- What ultimately made you decide to sign?
- Who is currently the primary economic source in your family? Did you consider economic factors when deciding to sign ACP?
- Did you experience external control (family members thinking I should do this) or internal pressure (feeling that they want me to make this decision) when making choices?
  - What is your role in the family/society, and how does it influence your decision-making?
  - What factors do you consider when making decisions?
  - Which factors can you control and which can't?
- What role did your religious beliefs play in the decision-making process of signing the ACP?

**Objective Three:**

- During the process, how did you participate in the discussion?
- During discussions with the medical team, which part do you think is most important?
- Do you think you received the medical care that aligns with your wishes? Please give examples.
- How can the healthcare team improve?
- Based on your observation, how has medical care changed after the implementation of the Patient Autonomy Act?

Thank you for taking the time to share your views and experiences with me.

**End Recording**
