# Supplementary material for: Exploring the Social and Cultural Influences on Advance Care Planning Engagement for Patients Living With Cancer: A Hermeneutic Phenomenology Study
Source: Nurs Inq. 2026 May 17;33:e70109. doi: 10.1111/nin.70109 (PMC13180463; doi:10.1111/nin.70109)
Supplement: Supplementary file 2 — Supporting File 2 [file NIN-33-e70109-s002.docx]

**Supplemental Information. Interview guide for family members**

**Interview Objectives**

1. Experiences and feelings when accompanying the patient in participating in medical decision-making.
2. Subjective obstacles and influencing factors that affect the patient's autonomous decision-making and interaction experiences.

**Objective One: Experiences and Feelings During ACP Participation**

- Can you start by sharing the process of [Patient's Name] falling ill?
- Are end-of-life issues openly discussed at home, or are there any reservations?
- What are your thoughts and values regarding ACP?
- How did participating in ACP make you feel?
  - Sad? Unacceptable?
  - Happy?
  - Has there been any part of the medical decision-making process that particularly impressed you?
  - If you were to share your experiences and feelings about participating in ACP with others, what would you share?
  - At what point did you discuss with doctors and family members?
  - How did participating in ACP change or impact your life?
- Based on your experience, how has the Patient Autonomy Act affected your right to medical decision-making? What is your opinion?
  - Do you think the law helps you exercise your medical autonomy?
  - What are the helpful aspects and limitations?

**Objective Two: Subjective Obstacles and Influencing Factors Affecting Patient's Autonomous Decision-Making**

- What made you willing to participate in signing [Patient's Name] ACP?
- What factors do you think would help [Patient's Name] make medical decisions?
- What factors do you think would hinder [Patient's Name] from making medical decisions independently?
- Did your religious beliefs influence you during the discussion with [Patient's Name]?
- What assistance do you think [Patient's Name] needs when making medical decisions?
- Can you share the thoughts discussed with [Patient's Name] before signing ACP?
  - What are the opinions of other family members?
  - Which family members opposed or agreed, and for what reasons?
  - What ultimately made you decide to support [Patient's Name] in signing?
- Who is currently the primary economic source in your family? Did you consider economic factors when deciding to sign ACP?
- Did you experience external control (family members thinking I should do this) or internal pressure (feeling that they want me to make this decision) when making choices?
  - What is your role in the family/society, and how does it influence your decision-making?
  - What factors do you consider when making decisions?
  - Which factors can you control and which can't?

**Objective Three**

- How did you participate in the discussion of [Patient's Name] ACP?
- During discussions with the medical team, which part do you think is the most important?
- Do you think the medical staff provided the medical care that [Patient's Name] wanted according to their needs?
  - (Probe: Yes...what was the process like? No...why not?)
- Did the medical team seek your opinion during the discussion? What was the process like?
- How can the healthcare team improve?
- Based on your observation, how has medical care changed since the implementation of the Patient Autonomy Act?
- Do you think the medical team now pays more attention to the medical decision-making rights of patients and their families?
  - Yes, please share your experiences and the process.
  - No, why not? What was your experience?

Thank you for taking the time to share your views and experiences with me.

**End Recording**
