# Supplementary material for: Exploring the Social and Cultural Influences on Advance Care Planning Engagement for Patients Living With Cancer: A Hermeneutic Phenomenology Study
Source: Nurs Inq. 2026 May 17;33:e70109. doi: 10.1111/nin.70109 (PMC13180463; doi:10.1111/nin.70109)
Supplement: Supplementary file 3 — Supporting File 3 [file NIN-33-e70109-s003.docx]

**Supplemental Information 3. Interview guide for healthcare professionals**

**Interview Objectives**

1. To provide experiences of patient ACP processes and factors affecting patient autonomous decision-making (socio-cultural context factors).
2. To share experiences and challenges of assisting patients in autonomous medical decision-making in clinical care.

**Objective One: Experiences of Patient ACP Processes and Factors Affecting Patient Autonomous Decision-Making (Socio-Cultural Context Factors)**

- Can you share your past experiences with executing ACP?
- Is there a difference in attitude towards ACP between those with better economic status and those less affluent?
- Do you think there is a correlation between educational level and signing ACP?
- Can you share your observations on the attitudes of different religious beliefs towards ACP? (e.g., Buddhism, Taoism, Christianity, etc.)
- Do most patients consider ACP as a personal choice or a family decision during the signing process?
- What factors do you think influence patients' willingness to sign ACP?
- What age range do most signatories fall into? (Ask using age brackets)
  - □ 20-29 years
  - □ 30-39 years
  - □ 40-49 years
  - □ 50-59 years
  - □ 60 years and above

**Objective Two: Experiences and Challenges of Assisting Patients in Autonomous Medical Decision-Making in Clinical Care**

- What motivated you to join the ACP consultation team initially?
- During the assistance process, who most often participate in the patient's autonomous medical decision-making based on your observation?
- What kind of patients have impressed you during your assistance?
- What kind of support do you provide when assisting patients in signing autonomous medical decisions?
- How do you handle situations where family members and patients have differing opinions during the assistance process?
- What reasons might lead patients to sign both DNR and ACP?
- Based on your experience, how has the Patient Autonomy Act affected the right to medical decision-making? What is your opinion?
  - Do you think the law helps patients exercise their medical autonomy?
  - What are the helpful aspects and limitations?
- Based on your observation, how has medical care changed since the implementation of the Patient Autonomy Act?
- Do you think the medical team now pays more attention to the medical decision-making rights of patients and their families?
  - Yes, please share your experiences and process.
  - No, why not? What was your experience?

**Objective Three:**

- What do you think are the biggest challenges in promoting ACP currently? What methods could improve promotion and public awareness?
- Have you signed an ACP yourself? Why or why not?

Thank you for taking the time to share your views and experiences with me.

**End Recording**
