# Supplementary material for: Exploring the Social and Cultural Influences on Advance Care Planning Engagement for Patients Living With Cancer: A Hermeneutic Phenomenology Study
Source: Nurs Inq. 2026 May 17;33:e70109. doi: 10.1111/nin.70109 (PMC13180463; doi:10.1111/nin.70109)
Supplement: Supplementary file 4 — Supporting File 4 [file NIN-33-e70109-s004.docx]

| **Supplemental Information 4. Sample characteristics of cancer patients, family caregivers, and healthcare professionals** | | | | | |
| --- | --- | --- | --- | --- | --- |
| **Cancer patients** | **n = 8** | **Family caregivers** | **n= 7** | **Healthcare professionals** | **n = 11** |
| Gender (male/female) | 2/6 | Gender (male/female) | 3/4 | Gender (male/female) | 3/8 |
| Age (years) |  | Age (years) |  | Age (years) |  |
| Mean average (SD) | 55.5 (27–76) | Mean average (SD) | 46.4 (23–63) | Mean average (SD) | 41.8 (29–56) |
| Diagnosis |  | Education level |  | Education level |  |
| Breast cancer | 3 | College | 7 | Bachelor | 6 |
| Lymphoma | 2 | Occupation / Profession |  | Master | 4 |
| Prostate cancer | 1 | Civil servant | 1 | Nursing diploma | 1 |
| Ovarian cancer | 1 | Service industry | 1 | Occupation / Profession |  |
| Leukemia | 1 | Commercial industry | 1 | Physician | 6 |
| Education level |  | Retired | 3 | Nurse | 3 |
| College | 5 | Nursing | 1 | Psychologist | 1 |
| High school | 2 | Religion |  | Social worker | 1 |
| Elementary school | 1 | Buddhism | 3 | Religion |  |
| Occupation / Profession |  | None | 4 | Buddhism | 2 |
| Retired | 3 | Marital status |  | Christianity | 4 |
| Homemaker | 2 | Married | 3 | Catholicism | 1 |
| Student | 1 | Divorced | 3 | None | 4 |
| Commercial industry | 2 | Single | 1 | Years of experience (HCPs) | Mean = 14.1 (range 1–30) |
| Religion |  | Living arrangement |  | Frequency of ACP participation |  |
| Buddhism | 5 | Nuclear family | 6 | >20 times | 9 |
| Taoism | 1 | Three-generation family | 1 | 11-15 times | 1 |
| None | 2 | Relationship to patients |  | 6-10 times | 1 |
| Marital status |  | Spouse | 2 |  |  |
| Married | 5 | Son or Daughter | 5 |  |  |
| Widowed | 1 |  |  |  |  |
| Single | 2 |  |  |  |  |
| Living arrangement |  |  |  |  |  |
| Nuclear family | 6 |  |  |  |  |
| Three-generation family | 1 |  |  |  |  |
| Independent | 1 |  |  |  |  |

ACP: Advance Care Planning
